# Nontuberculous Mycobacteria Infections in Children: A Clinical Overview of Diagnosis and Management

**DOI:** 10.3390/microorganisms14010130

**Published:** 2026-01-07

**Authors:** Alessandra Li Pomi, Antonella Gambadauro, Francesca Galletta, Giuseppe Fabio Parisi, Salvatore Leonardi, Pietro Sciacca, Milena La Spina, Sara Manti

**Affiliations:** 1 Pediatric Unit, Department of Human Pathology in Adult and Developmental Age ‘Gaetano Barresi’, University of Messina, 98124 Messina, Italy; gambadauroa92@gmail.com (A.G.); francygall.92@gmail.com (F.G.); 2 Pediatrics Unit and Pediatric Emergency Unit, AOU “Rodolico-San Marco”, “San Marco” Hospital, University of Catania, 95124 Catania, Italy; milena.laspina@unict.it; 3 Pediatric Respiratory Unit, Department of Clinical and Experimental Medicine, “San Marco” Hospital, University of Catania, 95121 Catania, Italy; gf.parisi@policlinico.unict.it (G.F.P.); leonardi@unict.it (S.L.); pietrosciacca@hotmail.it (P.S.)

**Keywords:** non-tuberculous mycobacteria, atypical mycobacteria, infection, paediatric age, children, child

## Abstract

Nontuberculous Mycobacteria (NTM), often referred to as environmental or atypical mycobacteria, are opportunistic pathogens phylogenetically as well as clinically distinct from both the *Mycobacterium tuberculosis* complex and *Mycobacterium leprae*. In the pediatric age group, NTM disease manifests with a diverse range of clinical phenotypes. Cervicofacial lymphadenitis stands out as the most common presentation among children who are immunocompetent. Conversely, skin and soft tissue infections, pulmonary disease and disseminated infections constitute less prevalent, yet clinically important, disease forms. Accurate identification is paramount, as differentiating NTM infections from tuberculosis (TB) remains challenging based solely on clinical symptoms, initial laboratory analyses, or standard radiological findings. This distinction is critical because treatment protocols for NTM infections differ substantially from those for tuberculosis. This narrative review offers a comprehensive and up-to-date summary of NTM infections in children. It examines the spectrum of clinical presentations and their prevalence, addresses the complexities of diagnosis and therapy, and underscores the importance of differential diagnosis against tuberculosis. Furthermore, we explore current diagnostic strategies, available therapeutic options, and the link between specific clinical syndromes and tailored management, pointing out existing knowledge gaps and suggesting priorities for future research. The absence of rapid, species-specific diagnostic tools often results in delayed initiation of targeted treatment, while overlapping clinical features with TB can lead to misdiagnosis. Therapeutic management is complicated by the necessity for prolonged drug courses, frequent occurrences of drug intolerance, limited availability of child-appropriate formulations, and the rising tide of antimicrobial resistance. Successfully tackling these issues demands enhanced surveillance, precise species-level identification, the creation of child-friendly drug formats, and the development of evidence-based treatment guidelines specifically designed for the pediatric population.

## 1. Introduction

Non-tuberculous mycobacteria (NTM), also referred to as atypical mycobacteria, encompass over 100 species within the genus Mycobacterium. These organisms are phylogenetically and clinically distinct from members of the *Mycobacterium tuberculosis* complex and from *Mycobacterium leprae* [1,2]. NTM are environmental opportunistic pathogens. They are ubiquitous in natural and man-made reservoirs, including soil, water sources, bioaerosols, dust, and a variety of animal hosts, both domestic and wild [1,2]. Importantly, there is no evidence to date of human-to-human transmission [2]. Despite their widespread distribution, NTM infections remain relatively uncommon in the general population.

In the pediatric population, NTM disease presents with variable clinical phenotypes. Cervicofacial lymphadenitis is the most frequent presentation in immunocompetent children, whereas skin and soft tissue infections, pulmonary involvement (often in the context of pre-existing chronic lung disease), and disseminated infections (primarily in patients with underlying immunodeficiencies) represent less common but clinically significant forms of disease [2,3].

Overall, NTM and *Mycobacterium tuberculosis* share several microbiological characteristics and exhibit overlapping immunological responses and clinical manifestations, particularly in lymphatic and pulmonary disease [3]. As a result, distinguishing NTM infections from tuberculosis based solely on clinical features, initial laboratory findings (including tuberculin skin testing) or radiographic imaging is often challenging [3]. Accordingly, molecular diagnostic techniques are essential for species-level identification [1]. Accurate diagnosis is critical to ensure the initiation of appropriate therapy, as treatment regimens for NTM infections differ significantly from those for tuberculosis [3].

This review provides a comprehensive and updated overview of non-tuberculous mycobacterial (NTM) infections in the pediatric population. It examines the spectrum of clinical presentations and their prevalence, discusses the challenges in diagnosis and treatment, and emphasizes the importance of differentiating NTM infections from tuberculosis. We also explore current diagnostic approaches, available treatment strategies, and the correlation between clinical syndromes and targeted therapy, highlighting the gaps in knowledge and proposing priorities for future research.

## 2. Search Strategy

### Methodology

The methodology of this review involves several key components aimed at ensuring an overview of clinical challenges and up-to-date analysis of the current research landscape. The literature included was identified by 3 independent investigators (A.L.P., A.G. and S.M.) principally using an automated literature search for English language papers published from January 2000 to August 2025. To capture the latest developments and to generate a wide search, a literature search of scientific databases such as PubMed, Scopus, and Web of Science was conducted, using keywords “non-tuberculous mycobacteria”, “atypical mycobacteria”, “child”, “children”, “pediatric age”. In addition to the automated search, a manual search for other relevant publications was conducted of the bibliographies of papers identified automatically. Articles were selected based on their relevance, novelty, and contribution to the field of research. Clinical guidelines, review articles, and illustrative case series that provided insights into new therapeutic approaches or enhanced understanding of disease mechanisms were included. For each selected article, data extraction was performed to gather information on study design, patient population, interventions, outcomes, and key findings. Emphasis was placed on studies that reported on clinical efficacy, safety, and mechanistic insights of therapies. Each study included in the review was critically appraised using an adapted version of standard evaluation criteria, assessing the methodological rigor, validity, and potential biases. This appraisal aimed to ensure the reliability of conclusions drawn and to provide a balanced view of the current state of research. The findings from the selected articles were synthesized thematically to provide a coherent narrative of recent studies.

## 3. Results

### 3.1. Publications Included

An initial literature search, utilizing the previously mentioned keywords and encompassing studies involving the adult population, identified a total of 46 scientific articles. Following assessment of the titles and abstracts, 30 papers were excluded because they were not related to the subject and 16 were deemed eligible for inclusion based on their relevance to the scope of this review. Subsequent refinement of the search strategy, through the incorporation of pediatric age-specific keywords, yielded an additional 6 articles. Among these, a total of 4 papers were found to be duplicated and were therefore excluded, and the remaining 2 were excluded as they were not published in the English language. A manual search considering the bibliographies of the review articles retrieved 35 additional papers. The analyses of all the articles by reading the full texts resulted in the exclusion of 29 papers.

### 3.2. Microbiology and Epidemiology

There are more than 170 recognized species of NTM, of which approximately 100 have been implicated in human disease [1,2,4]. Their ability to persist in the environment and cause opportunistic infections is largely attributable to distinctive microbiological features. In particular, their highly hydrophobic cell wall—rich in mycolic acids—confers acid-fastness and enables resistance to chemical disinfectants and antibiotics [4]. NTM are ubiquitous environmental organisms, with a strong presence in soil and water, including natural water bodies, drinking water, and household plumbing. These microbes can become aerosolized within water droplets and persist in dust particles, which are abundant in high-moisture environments like sinks and showers. The lipid-rich outer membrane enhances adherence to surfaces and promotes survival in water systems and indwelling medical devices such as catheters. Moreover, NTM exhibit remarkable resilience under harsh environmental conditions, including high temperatures, acidic pH, and hypoxia [4]. As a result, aerosol inhalation from municipal and private water sources is considered the primary route of transmission to humans [5].

Based on their growth rate in culture, NTM species are widely divided into two groups: slow-growing mycobacteria (SGM), which need at least 8 days to create visible colonies, and rapid-growing mycobacteria (RGM), which usually grow in 3 to 7 days [1,4]. Certain species, like *Mycobacterium ulcerans* and *Mycobacterium genavense*, may need as long as 8 to 12 weeks to grow [4]. While NTM infections are uncommon among immunocompetent hosts, paediatric patients may acquire the infection following environmental exposure, for example, to swimming pools or natural ponds [3,6]. In the hospital setting, nosocomial NTM outbreaks have been associated with hospital water systems, affecting paediatric patients, especially those undergoing hematopoietic stem cell or bone marrow transplantation [7]. In addition, iatrogenic spread may be attributed to contaminated medical instruments or cosmetic procedures, including dental and surgical interventions [3,6]. A study demonstrated that children with cystic fibrosis (CF) living near aquatic environments had an elevated risk of NTM acquisition, with proximity within 500 m significantly increasing the likelihood of infection [8]. As a further source, soil serves as a known reservoir for NTM, frequently introduced into indoor spaces via domestic animals and footwear [3]. Despite these well-defined environmental sources, the specific transmission pathways for NTM are not yet completely elucidated [3]. Significantly, direct transmission between humans or between animals is deemed exceptionally rare [5].

The occurrence of NTM disease among children is rising globally [9]. The exact geographical distribution of NTM infections is difficult to establish due to the absence of standardized national surveillance systems [3,6]. While studies conducted in adult populations from high-income countries have reported an increase in NTM diagnoses over recent decades, equivalent data for paediatric populations are lacking. Furthermore, large-scale epidemiological studies have demonstrated considerable variation in incidence and species distribution across different countries and regions [3,6]. The true prevalence of NTM infections is frequently underreported in low-resource settings. Here, diagnosis often happens as a secondary finding while investigating potential cases of tuberculosis, particularly when acid-fast bacilli are identified in respiratory specimens [3,6]. A recent review has described the prevalence of NTM disease ranging from 0.6 to 5.36 cases per 100,000 across countries, with Europe showing the highest rates [9]. On the other hand, in a recent Spanish cohort study, case numbers stayed consistent throughout the initial prospective phase (2013–2020) but showed a marked decrease in 2021–2022. In 45.9% of cases, disease onset occurred in spring or in June. Moreover, a seasonal trend has been observed, with higher incidence reported in late winter and early spring [4].

A recent systematic review and meta-analysis has evaluated data from studies conducted in mainland China between 2000 and 2019 on the prevalence, species distribution, and antimicrobial susceptibility of NTM [10]. The crude isolation rate of NTM among patients suspected of tuberculosis ranged from 4.66% to 5.78%, accounting for 11.57% of all Mycobacterium isolates. *M. abscessus* and the *M. avium complex* (MAC) were the most frequent species, with *M. intracellulare* later emerging as the predominant species. NTM displayed limited susceptibility, mainly to ethambutol, linezolid, clofazimine, amikacin, tobramycin, and clarithromycin. Overall, the prevalence of NTM showed a decreasing trend, with marked geographic variability in species distribution and highly heterogeneous resistance patterns, underscoring the need for cautious empirical therapy and the development of more structured surveillance systems [10].

Similarly, an up-to-date review published in 2023 summarizes current evidence on the epidemiology, clinical features, diagnosis, and management of NTM infections in Africa [11]. Most data originated from South Africa, Ethiopia, and Nigeria. Reported prevalence varied from 0.2% to 28%. The predominant NTM species were MAC, *M. fortuitum*, and *M. abscessus*. NTM infections were frequently reported among HIV-positive individuals, with additional risk factors including older age, chronic lung disease, and prior tuberculosis. The review underscores the substantial burden and frequent misdiagnosis of NTM due to their clinical similarity to tuberculosis, alongside limited diagnostic resources across the continent [11].

Moreover, an observational study conducted in French Guiana investigated the epidemiology of pulmonary NTM involvement over the 2008–2018 decade, assessing the burden of these infections in the region [12]. Among 178 patients with at least one positive respiratory culture, 147 were classified as having casual Pulmonary NTM isolation and 31 as having NTM pulmonary disease. The estimated annual incidence was 6.17 per 100,000 inhabitants for respiratory isolates and 1.07 per 100,000 for the disease. MAC was the most frequently isolated species (38%), followed by *M. fortuitum* (19%) and *M. abscessus* (6%), the latter two predominantly identified in the coastal central region. NTM pulmonary disease was mainly associated with MAC (81%) and *M. abscessus* (16%). As the first epidemiological study on PNTM in French Guiana, it shows incidence rates comparable to other settings and underscores the need to consider NTM infections even in contexts with high tuberculosis prevalence [12].

In general, the most frequently identified species are *Mycobacterium avium* complex (MAC) (43.1%) and *Mycobacterium lentiflavum* (39.9%) [9,13]. Specifically, in adults, *Mycobacterium avium* is more prevalent than *Mycobacterium intracellular* within MAC isolates [9]. Unlike in the paediatrics population, MAC is the most commonly isolated pathogen [9]. Recently, in Bulgaria, a new species has been identified [14]. Following the SeqCode procedure, the designation *Mycobacterium bulgaricum* sp. nov. was proposed. The shifting prevalence of NTM species in Bulgaria between 2011 and 2022 reflects the emergence of novel species and variations influenced by environmental and demographic factors. These findings emphasize the critical role of precise species identification and genotyping in advancing the understanding of NTM epidemiology, guiding public health interventions, and improving diagnostic precision and therapeutic approaches [14]. Whether the observed rise in NTM disease represents a genuine increase in incidence or is instead the result of improved diagnostic capabilities remains unclear [4]. The expanded use of molecular techniques, enhanced clinician awareness, and improved laboratory infrastructure may all contribute to higher detection rates. Additionally, the growing population of immunosuppressed children, particularly those receiving immunomodulatory or immunosuppressive therapies, may be contributing to the apparent increase in NTM cases [4].

### 3.3. Immunology

In immunocompetent individuals, NTM are generally considered commensal or environmental organisms, as the host immune system is typically capable of containing and eradicating the infection through both innate and adaptive immune responses [3]. Immune defense against NTM involves a collaborative action of T lymphocytes, natural killer (NK) cells, and macrophages. A recent molecular study conducted in China has highlighted the involvement of specific host proteins in the activation of macrophage-mediated immune responses against NTM [15]. In this context, experimental evidence has shown that *M. abscessus* and *M. smegmatis* promote M1 macrophage polarization, characterized by enhanced nitric oxide production and pro-inflammatory cytokine expression. This study identified the non-histone nuclear protein HMGN2 as a key regulator of this process, acting as a negative modulator of M1 polarization through NF-κB and MAPK signaling pathways. Silencing HMGN2 enhanced macrophage antimicrobial activity and reduced intracellular NTM survival, supporting its role in the regulation of innate immune responses during NTM infection [15].

Macrophages are pivotal in immune defense against NTM by producing interleukin IL-12, which stimulates the release of interferon-gamma (IFN-γ) from T cells and NK cells. IFN-γ, in turn, potently activates macrophages, bolstering their capacity to eliminate intracellular pathogens. Consequently, individuals—particularly in the paediatric population—with impairments in T cell immunity (e.g., Human Immunodeficiency Virus-HIV/Acquired Immune Deficiency Syndrome-AIDS) or the IL-12/IFN-γ signalling axis are at an elevated risk of severe or disseminated NTM infections [4]. Fortunately, due to the widespread availability of antiretroviral therapy, children living with HIV and severely depressed CD4+ T-cell counts are now rare, resulting in a significant reduction in the incidence of disseminated NTM disease in this population [3]. Among primary immunodeficiencies, Mendelian Susceptibility to Mycobacterial Disease (MSMD) is a rare genetic syndrome associated with impaired immunity against mycobacteria, and involving macrophage–T helper cell interactions. MSMD is caused by mutations in components of the IL-12/23–IFN-γ pathway, most commonly affecting the IL-12 receptor β1 subunit (IL12RB1) or the IFN-γ receptor 1 (IFNGR1) and resulting in a defective macrophage activation and inadequate clearance of mycobacteria [3,16]. Another mechanism contributing to susceptibility is the acquired development of autoantibodies against IFN-γ, which neutralize its activity and impair the host’s mycobacterial defense [3]. In addition to immunodeficiencies, other non-immunological conditions are associated with increased NTM risk, including cystic fibrosis (CF), non-CF bronchiectasis, α1-antitrypsin deficiency, and structural lung abnormalities [4]. Finally, iatrogenic factors, such as the use of central venous catheters, and patients undergoing solid organ or hematopoietic stem cell transplantation, are at increased risk of disseminated NTM infections due to their compromised immune status and repeated exposure to potential sources of infection [3,4].

### 3.4. Clinical Features of Atypical Mycobacteria Infection

#### 3.4.1. Lymphadenitis

Lymph nodes are the most frequently affected site, followed by the lungs and skin or soft tissue. Lymphadenitis is the most frequent clinical manifestation of NTM disease in the pediatric population [2]. The estimated incidence ranges from 0.8 to 3.5 cases per 100,000 children, occurring predominantly in otherwise healthy children aged one to five years, and typically involving the cervicofacial region [2,17]. Recent data suggest a potential increase in incidence in specific geographical areas: an annual incidence of 4.7 cases per 100,000 children younger than five years of age has been recently reported in Cyprus [2].

The main portal of entry is believed to be the oral cavity, via the oropharyngeal mucosa, although skin lesions may also serve as an entry point for the pathogen [2].

NTM lymphadenitis most commonly involves the preauricular, submandibular, and cervical lymph nodes. Less commonly, posterior cervical nodes may also be involved [17]. Clinically, children typically present with unilateral, painless, and slowly progressive lymph node enlargement, often involving one or more nodes. Over several weeks—typically after one month—the overlying skin becomes progressively thinner and violaceous, sometimes leading to fistula or sinus tract formation with drainage of purulent material [17,18]. In particular, preauricular involvement carries a risk of facial nerve injury, either due to local inflammation or as a complication of surgical intervention [18]. In most cases, NTM lymphadenitis remains localized to the affected lymph nodes, and systemic symptoms are typically absent. Children usually appear well and do not present with constitutional signs such as fever, weight loss, or malaise [2,17]. The most frequently isolated pathogen in pediatric NTM lymphadenitis is the MAC, including several species such as *Mycobacterium hemophilum*, *Mycobacterium Intracellulare* and *Mycobacterium scrofulaceum* [2,13,17,19,20].

#### 3.4.2. Cutaneous and Soft Tissue Disease

Cutaneous infections caused by NTM can present with a broad spectrum of clinical manifestations, including cellulitis, persistent ulcers, subacute or chronic nodular lesions, and abscesses [4,18]. These infections typically result from direct inoculation into compromised skin, commonly through skin abrasions, open wounds, fractures, surgical or injection sites, puncture injuries (e.g., stepping on a nail), or environmental trauma (such as combat-related injuries with contamination) [4]. NTM skin infections primarily affect otherwise healthy individuals and are most frequently caused by RGM, such as MAC, *Mycobacterium fortuitum*, and *Mycobacterium chelonae* [17,21]. These species are often associated with painful abscess formation and drainage at the site of infection. However, cutaneous involvement in immunocompromised patients may represent a manifestation of disseminated disease, thus requiring a comprehensive systemic evaluation [4].

In recent years, RGM-related infections have been increasingly reported following cosmetic procedures performed in developing countries, often due to breaches in sterile techniques and exposure to contaminated water. A notable multistate outbreak in the United States in 2013 involved 21 individuals who developed NTM infections after undergoing cosmetic surgeries in the Dominican Republic. The most isolated organisms were *Mycobacterium abscessus* and *Mycobacterium fortuitum* [4]. Clinical presentations, in such cases, included deep or superficial wound infections, tender erythematous nodules, pustules, and granulomatous lesions around incision sites, often with wound dehiscence and purulent drainage, though systemic symptoms were generally mild or absent [4]. A distinct clinical entity is the Buruli ulcer, a slow-growing NTM classified as a neglected tropical disease and caused by *Mycobacterium ulcerans*. This infection is endemic in West and Central Africa and in southeastern Australia, with a particular predilection for pediatric population [4,17]. Buruli ulcer typically begins as a painless papule or nodule on the limbs, which can progress to extensive ulceration, occasionally involving the head, neck, or genital region. If left untreated, it can lead to severe tissue necrosis, limb contractures, scarring, and disfigurement. Notably, antimicrobial treatment may initially worsen clinical symptoms due to a paradoxical inflammatory reaction, before clinical improvement occurs [4].

#### 3.4.3. Pulmonary Disease

Pulmonary disease caused by NTM represents a distinct clinical entity, differing substantially from other NTM-related syndromes. It is almost invariably associated with preexisting pulmonary conditions, most notably CF- bronchiectasis, but also occurs in children with non-CF bronchiectasis, primary ciliary dyskinesia, primary immunodeficiencies, and those with heterozygous CFTR mutations without overt CF [4,18]. While deficiencies in the IL-12–IFNγ axis increase susceptibility to pulmonary NTM disease, interestingly, other immunodeficiencies strongly linked to tuberculosis—such as chronic granulomatous disease, severe combined immunodeficiency (SCID), and DiGeorge syndrome—do not appear to predispose to pulmonary or disseminated NTM infections [18]. The most isolated organisms in pediatric pulmonary NTM infections are members of the MAC, followed by MAC and *Mycobacterium chelonae*, with rapidly growing mycobacteria more frequently encountered in younger children. The relative prevalence of these species varies by age and geography [4,18]. NTM pulmonary disease is rare in otherwise healthy children, but it is frequently described in lung transplant recipients. Notably, outbreaks of NTM infection within CF centers have raised concern about potential nosocomial or environmental transmission [4].

The clinical presentation of pulmonary NTM infection is nonspecific, often mimicking exacerbations of underlying lung disease. Symptoms may include worsening cough, increased sputum production, decline in pulmonary function, fatigue, weight loss, and fever. In children with CF or other structural lung disease, such changes may signal a superimposed NTM infection [4,18]. There are no specific radiological findings. In CF patients, small pulmonary nodules with bronchiectasis are most common. A tree-in-bud pattern, reflective of bronchiolar involvement, is frequently seen on CT scans. Additional radiologic presentations include nodular or cavitary opacities, fibrocavitary lesions (which may mimic tuberculosis), and large solitary nodules that could raise suspicion for malignancy. These findings are not specific to NTM and overlap with other infectious and inflammatory diseases [4,18]. A unique radiologic and clinical manifestation is hypersensitivity pneumonitis, or “hot tub lung”, observed in immunocompetent children and adults after exposure to aerosolized NTM from contaminated hot tubs or spa pools. This condition is thought to represent an exaggerated immune response rather than true infection. Imaging typically reveals extensive ground-glass opacities, centrilobular nodularity, and consolidation [4].

#### 3.4.4. Disseminated Disease

Disseminated NTM disease in children is rare, and its occurrence strongly suggests underlying immunodeficiency. The most implicated pathogen is MAC, responsible for over 90% of disseminated cases [4,18].

The clinical features are non-specific, often including fever, weight loss, lymphadenopathy, cutaneous lesions, gastrointestinal symptoms, and occasionally pulmonary or soft tissue infections. In immunocompromised children, skin involvement may represent an early manifestation. Disseminated NTM infections have been described in association with primary immunodeficiencies, hematologic malignancies, organ transplantation, and advanced HIV infection. During the AIDS epidemic, the incidence of disseminated MAC rose dramatically, but has since decreased with the widespread use of highly active antiretroviral therapy (HAART) [4].

NTM-associated Reconstitution Inflammatory Syndrome (IRIS) is a paradoxical inflammatory reaction that occurs upon restoration of immune function, as seen after initiating HAART in HIV-positive children or following a reduction in immunosuppression in transplant recipients. IRIS may occur with or without a prior diagnosis of NTM infection and typically presents within weeks to months of immune reconstitution. Clinical features include fever, night sweats, lymphadenitis (peripheral, thoracic, or abdominal), pulmonary infiltrates, and hepatosplenomegaly [4].

NTM, particularly such as *Mycobacterium mucogenicum*, *Mycobacterium fortuitum*, and *Mycobacterium abscessus*, have been implicated in catheter-associated bloodstream infections [22]. These infections occur in both immunocompetent and immunocompromised patients, including those with hematologic malignancies. Patients typically present with fever, although neutropenia may be absent. NTM may also cause exit site infections, peritonitis in children on peritoneal dialysis, or meningitis in those with ventriculoperitoneal shunts [4,22].

**Figure 1:**
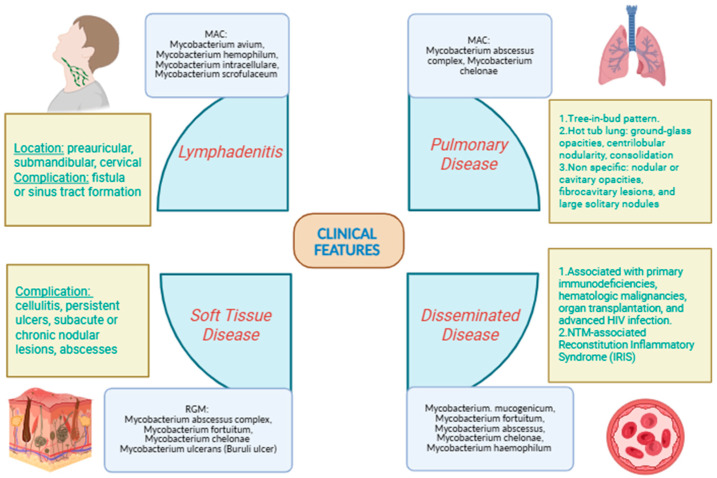
Summary of the main clinical syndromes associated with non-tuberculous mycobacterial (NTM) infection in various body sites and the corresponding primary aetiology.

The main clinical presentation patterns of NTM disease in various body sites and the primary responsible pathogens are summarized in Figure 1.

### 3.5. Diagnosis of Atypical Mycobacteria Infection

Diagnosis of NTM disease requires clinical, microbiological, and radiological assessment [3]. Confirmation is achieved by PCR or culture, ideally using both solid and liquid media at varied incubation temperatures [20]. In recent years, several studies have investigated the use of PCR-based assays for the identification of NTM. A recent study conducted in Ecuador evaluated the performance of two commercial PCR kits for the identification of Mycobacterium tuberculosis complex and NTM in a high-burden setting [23]. Both assays achieved 100% sensitivity for *M. tuberculosis*, while one kit showed superior sensitivity for NTM detection (94.9%) compared with the other (77.1%), supporting its reliability for the rapid discrimination between *M. tuberculosis* and NTM in clinical isolates [23].

Culture identification distinguishes NTM from *Mycobacterium tuberculosis* at low cost. Drug susceptibility testing remains limited, meaning that the ability to determine how effectively different drugs can treat a particular infection or disease is still restricted due to technological, logistical, or resource-related challenges [3]. NTM lymphadenitis in children is usually paucibacillary and often diagnosed presumptively without microbiological confirmation. Bacterial culture has limited sensitivity (41–80%), whereas molecular detection in lymph node biopsies is more sensitive (up to 72%), particularly valuable for pathogens like *Mycobacterium haemophilum* that are hard to culture or in settings limited to routine solid media at 37 °C. Fine needle aspirates outperform biopsies for microbiological diagnosis by PCR or culture, but excision biopsy can be both diagnostic and curative. Some NTM species grow best at ~30 °C, so cultures should be incubated at both 30 °C and 37 °C. For optimal detection of *Mycobacterium haemophilum*, media must be enriched with an iron source such as hemin or ferric ammonium citrate [3]. The preferred approach to the diagnosis of NTM adenitis is excision of the affected node, because incision and drainage can be complicated by fistula formation. Pathologic examination of affected tissue often reveals Acid-fast Bacillus (AFB) with caseating or noncaseating granulomas. However, staining and pathology studies do not distinguish between disease due to NTM and *Mycobacterium tuberculosis*. In a 10-year review of NTM cases, the greatest diagnostic yield was observed with PCR testing of lymph node material (91.3% sensitivity), followed by culture (64.8% sensitivity) and microscopy for AFB (30.3% sensitivity) [4].

Recent research has also focused on NTM as emerging pathogens in surgical wound infections [24]. In this context, a changing microbial profile of surgical site infections has been reported, with NTM increasingly recognized as potential pathogens. A recent study analyzing 192 pus samples from SSI cases identified mycobacterial infections in a small proportion of patients, including both *M. tuberculosis* and NTM. Species-level identification using matrix-assisted laser desorption ionization–time of flight (MALDI-TOF) mass spectrometry revealed several NTM species, whereas PCR-based microarray showed limited concordance. Although the prevalence of NTM was low, these findings emphasize the importance of considering NTM in surgical site infections diagnostics and support the use of advanced identification techniques to guide appropriate treatment [24].

Moreover, a cross-sectional study conducted in Iran analyzed 45 sputum-derived mycobacterial isolates using molecular sequencing of the 16S rRNA and *rpoB* genes to assess species diversity [25]. NTM were identified in 83% of samples, with a wide range of species detected, including *M. simiae*, *M. fortuitum*, and *M. abscessus* as the most prevalent. Sequencing of the *rpoB* gene showed high discriminatory power, supporting the combined use of 16S rRNA and *rpoB* sequencing as a reliable approach for accurate NTM identification in clinical samples [25].

The diagnosis of NTM-associated pulmonary disease requires compatible clinical, radiologic, and microbiological evidence, with exclusion of alternative diagnoses [3,22]. In adults and older children, ≥3 early morning sputum cultures are recommended, while in patients with CF two positive cultures are generally required. Bronchoalveolar lavage or induced sputum may also be used in diagnosing. The disease is rare in children, and gastric aspirates are discouraged due to frequent clinically irrelevant isolates. Respiratory samples should be decontaminated with 1% N-acetyl-L-cysteine–sodium hydroxide, and in CF or Pseudomonas-colonized samples, an additional 5% oxalic acid step can improve sensitivity. Combining liquid and solid cultures increases yield from 66% and 51%, respectively, to 76% [3]. Diagnosing pulmonary NTM disease remains challenging, especially in the pediatric population. It is often difficult to determine whether the isolated NTM represents true infection, colonization, or environmental contamination. To assist with diagnosis, consensus criteria incorporating clinical, microbiologic, and radiologic findings have been developed, though these have not been clinically validated in pediatric populations, especially in children with CF [18]. A positive culture alone does not confirm disease, and prolonged follow-up with multiple confirmatory samples is often needed [3,4]. Diagnostic criteria—established for adults, especially with *Mycobacterium avium*, *Mycobacterium kansasii*, and *Mycobacterium abscessus*—may not apply to children or other species. Differentiating NTM disease from tuberculosis is particularly challenging in high-TB-burden settings, where empirical TB treatment is rated while waiting for NTM identification. The clinical presentation included older age, fever, weight loss, reduced tuberculin reactivity, and fewer supportive radiographic findings compared with TB. Pediatric data are limited [3].

Disseminated NTM disease is usually confirmed by positive AFB blood culture using bottles specifically intended for mycobacteria isolation, or by culture of bone marrow biopsy specimens, both offering similar sensitivities ranging from 60 to 80% [3,4]. Because bacteremia may be intermittent, multiple blood cultures should be obtained. Staining and culture of liver biopsy specimens can provide a faster and slightly more sensitive alternative to blood and bone marrow culture. Automated systems for mycobacterial blood culture—each with a sensitivity of about 80%—are preferred; in settings without such systems, lysed and centrifuged samples should be incubated in both liquid and solid media, as omission of one medium decreases sensitivity by 10–15%. Cultures from other sites, including lymph nodes, may also aid in diagnosis [3,22]. NTM bloodstream infection is mainly seen in oncologic patients with central venous catheter [26]. Disseminated skin disease due to rapid-growing mycobacteria such as *Mycobacterium abscessus*, *Mycobacterium chelonae*, and *Mycobacterium haemophilum* typically affects patients with haematological malignancies or solid organ transplants, but rarely those with HIV/AIDS [4,22]. Diagnosis is usually made by culture of skin biopsy specimens. Although sensitivities and specificities of bacterial culture and PCR for these samples have not been systematically studied, incubation at both 30 °C and 37 °C is advised—particularly for detecting *Mycobacterium haemophilum* in tuberculosis-endemic areas and *Mycobacterium marinum* [3].

### 3.6. Treatment of Atypical Mycobacteria Infection

To provide both definitive treatment and an etiological diagnosis, through histopathological and microbiological examination, the standard therapeutic approach to NTM lymphadenitis in children was complete surgical excision of the selected lymph nodes for many years [2,20]. When complete excision was not feasible—due to anatomical constraints or risk of complications—antimicrobial therapy was considered the preferred alternative [2,20]. More recently, several studies have proposed a paradigm shift in the management of NTM lymphadenitis. Emerging evidence supports the use of antimycobacterial antibiotic therapy as first-line treatment, or even watchful waiting without intervention in selected cases, particularly when the disease is mild and self-limited [26]. However, when relying solely on medical therapy, a definitive etiological diagnosis is often lacking, as PCR and other microbiological analyses typically needs surgical specimens for confirmation [22]. Zimmermann et al. showed that fewer than 20% of pediatric patients were managed with antibiotic therapy or observation, with surgery remaining the most reported treatment modality [17]. Among surgical interventions, complete excision was the most frequently employed and demonstrated an adjusted mean cure rate of 98%. However, the most notable complication was facial nerve palsy, reported in 10% of patients, although it was permanent in only 2% [17]. In contrast, incision and drainage were associated with a lower pooled cure rate of 34%, a non-negligible recurrence rate, and complications such as fistula formation and poor cosmetic outcomes [17]. Curettage and fine-needle aspiration are less commonly adopted in the management of NTM infections because these procedures often fail to achieve complete removal of infected tissue. As a result, they are associated with a higher risk of residual disease, persistent local swelling, and lower overall cure rates when compared with complete surgical excision, which remains the preferred approach in appropriately selected cases. Incomplete excision, described in only four publications, was linked to the persistence of skin sinuses for several months [7]. On the other hand, clinical observation and antibiotic treatment showed similar mean cure rates of approximately 70% [17]. Observation alone was reported in nine studies, with spontaneous resolution in approximately 90% of cases, suggesting that conservative management may be appropriate in selected patients with mild, non-progressive disease [17]. However, the evidence base remains limited, due to the lack of well-designed RCTs in guiding therapeutic decision-making [17]. In some cases, antibiotic treatment was used in combination with surgery, either preoperatively to reduce inflammation or postoperatively to prevent recurrence [17].

The most used antibiotic regimen for NTM lymphadenitis includes clarithromycin, often in combination with rifampicin, rifabutin, or ethambutol. Macrolide monotherapy might be considered. The studies reviewed reported variable dosing of clarithromycin, dose range 15 to 30 mg/kg/day, with treatment durations ranging from 1 to 52 weeks, also reflecting significant heterogeneity in clinical practice. Medical therapy can be associated with adverse effects. The most frequently reported was reversible tooth discoloration, followed by fever and fatigue [17].

Regarding skin and soft tissue disease, while uncomplicated cutaneous NTM infections may resolve spontaneously, treatment decisions should be based on the specific NTM species involved, the extent of disease, and the immune status of the host. Antimycobacterial therapy and/or surgical intervention are often recommended to accelerate resolution and prevent long-term sequelae [4,18]. Treatment usually involves surgery plus prolonged antibiotic therapy targeted to the isolated species [3]. *Mycobacterium abscessus* infections are challenging due to resistance; depending on subspecies, clarithromycin, amikacin, and tigecycline show good in vitro activity [3]. Suggested regimens include macrolide, amikacin, and cefoxitin or imipenem, together with surgical debridement [3]. Therapy should last at least four months, with a minimum of two weeks of intravenous treatment [3].

For pulmonary disease, treatment decisions are complex and must be personalized, balancing the uncertain benefits of therapy with the drug toxicity and burden of prolonged multidrug regimens. Therapy is based on species identification and macrolide susceptibility testing, since macrolides typically represent the cornerstone of treatment. Given the potential side effects and prolonged therapy duration, clinicians must carefully evaluate the clinical significance of the NTM isolate before initiating treatment [18]. Given resistance and toxicity issues, treatment should be supervised by specialists in mycobacterial infections [3,4]. For MAC pulmonary disease, the standard regimen includes a macrolide, ethambutol, and a rifamycin, with streptomycin or amikacin added for severe cases, continued until sputum cultures are negative for at least one year [3,4,27]. In infants, typical treatment regimens consisted of ethambutol combined with rifampin or clarithromycin, as well as clarithromycin paired with amikacin [28]. *Mycobacterium kansasii* disease is treated with rifampicin, isoniazid, and ethambutol, usually for one year, regardless of sputum conversion timing [3,27]. MAC lung disease requires an individualized regimen based on susceptibility testing, typically starting with a macrolide plus two parenteral agents (amikacin, cefoxitin, imipenem, or tigecycline) for several months, followed by a continuation phase with 2–3 oral agents for a total of 12 months after sputum culture conversion [3,27]. Adjunctive surgery may be considered for localized disease with poor drug response [3]. Clofazimine is increasingly explored as an oral option for pulmonary NTM [3].

In children with HIV, first-line treatment for disseminated MAC includes initial rifabutin to improve bacteremia clearance and survival, followed by macrolide and ethambutol administration [4]. Aminoglycosides may be used for breakthrough infections or suspected macrolide resistance [4]. After clinical improvement, maintenance therapy with at least two active drugs should continue for life or until immune restoration is achieved [4]. In other causes of cellular immunodeficiency, including Mendelian Susceptibility to Mycobacterial Disease (MSMD), the same regimens apply [4]. Some patients with MSMD respond to subcutaneous INF-γ plus antibiotics, though this is ineffective in complete IFN-γ receptor or STAT1 defects [4]. Hematopoietic stem cell transplantation has been attempted in severe MSMD with variable success [4].

**Figure 2:**
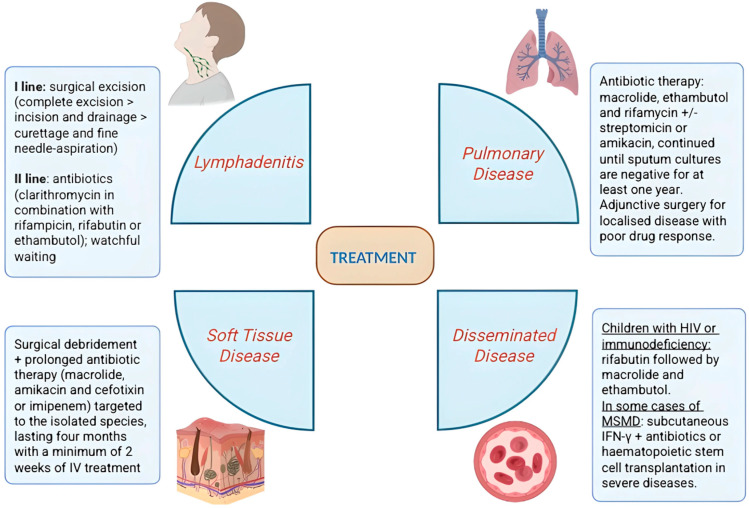
Treatment regimens for Non-tuberculous Mycobacterial (NTM) disease stratified by infection site. These data are general guidelines, not meta-analysis-based evidence, and that dosages must be adjusted individually.

The main therapeutic approaches for NTM disease, categorized by the involved body site, are summarized in Figure 2.

## 4. Discussion

NTM infections exhibit distinct epidemiological, clinical, and therapeutic patterns in adults compared with pediatric populations. In adults, NTM disease predominantly involves the lungs and is frequently associated with underlying structural lung disease, chronic respiratory conditions, or immunosuppression, often presenting as a slowly progressive pulmonary infection. Management typically requires prolonged, species-specific multidrug regimens, most commonly including macrolides, ethambutol and rifampicin, with treatment duration extending for at least 12 months after microbiological culture conversion [29].

In contrast, children more commonly develop extrapulmonary NTM infections, particularly cervical lymphadenitis in otherwise healthy hosts, while pulmonary disease remains relatively uncommon. In pediatric patients, complete surgical excision is often considered the treatment of choice for localized lymphadenitis and may be curative, whereas antimicrobial therapy is reserved for disseminated disease, incomplete resection, or immunocompromised children. When required, antibiotic regimens are generally shorter and carefully tailored to minimize drug-related toxicity [3]. These differences highlight the importance of age-specific diagnostic and therapeutic strategies in the management of NTM infections.

In recent years, growing interest has focused on novel therapeutic strategies for NTM infections in pediatric patients, aiming to improve treatment outcomes while limiting drug-related toxicity. Newer antimicrobial agents, such as liposomal amikacin for inhalation, have shown promise in refractory pulmonary NTM disease by enhancing local drug delivery and reducing systemic exposure. Oxazolidinones, including linezolid and tedizolid, and the re-emerging use of clofazimine have also been explored as adjunctive options, particularly in macrolide-resistant or disseminated infections. In parallel, host-directed therapies targeting macrophage activation and immune modulation are being investigated as potential adjuncts to antimicrobial regimens, with the goal of enhancing intracellular mycobacterial clearance. Although clinical evidence in children remains limited, these emerging approaches may represent valuable alternatives for complex or treatment-refractory pediatric NTM infections and warrant further prospective evaluation [29,30].

Despite growing recognition of NTM infections as clinically relevant pathogens, substantial gaps remain in the understanding of NTM disease in pediatric populations. Current evidence is largely derived from small retrospective series or extrapolated from adult studies, limiting the reliability of epidemiological estimates and the applicability of diagnostic and therapeutic strategies in children. Species-specific clinical behavior, age-related host–pathogen interactions and optimal diagnostic algorithms remain poorly defined. Moreover, the lack of pediatric pharmacokinetic and pharmacodynamic data for most antimycobacterial agents hampers dose optimization and increases the risk of adverse effects. Addressing these limitations will require coordinated prospective studies, pediatric-focused clinical trials, and the development of age-specific guidelines, which represent essential future directions to improve the management and outcomes of NTM infections in children.

## 5. Limitations Study

This is a narrative review and not a systematic analysis. Therefore, selection bias is possible. We prioritized studies that illustrate specific clinical phenotypes over a statistical synthesis of all available data.

## 6. Conclusions

NTM infections in children present significant clinical and public health challenges. Their increasing global prevalence, coupled with the wide spectrum of causative species and variable clinical presentations, complicates timely diagnosis. The lack of rapid, species-specific diagnostic tools often delays targeted treatment, while overlapping features with tuberculosis may lead to misdiagnosis. Prolonged treatment regimens, frequent drug intolerance, limited pediatric formulations, and the emergence of antimicrobial resistance hinder therapeutic management. In resource-limited settings, restricted access to essential drugs and specialized surgical expertise further worsens patient outcomes. Addressing these issues requires improved surveillance, species-level identification, development of child-friendly drug formulations, and evidence-based treatment protocols tailored to the pediatric population.

## Data Availability

No new data were created or analyzed in this study. Data sharing is not applicable to this article.
